# Adolescent boys’ experiences of mental health and school health services - an interview study from Norway

**DOI:** 10.1186/s12889-024-18952-6

**Published:** 2024-05-31

**Authors:** Tonje Helland Lindborg, Inger Kristensson Hallström, Astrid Synnøve Litland, Helene Åvik Persson

**Affiliations:** 1 Department of Health and Caring Science, Faculty of Health and Social Sciences, Western Norway University of Applied Sciences, Bergen, Norway; 2 Department of Health Sciences, Faculty of Medicine, Lund University, P.O. Box 157, Lund, SE 221 00 Sweden; 3 Department of Children and Families, Bergen Municipality, Bergen, Norway

**Keywords:** Adolescence, Boys, Experience, Empowerment, Help-seeking, Mental health literacy, Qualitative, School nurse

## Abstract

**Background:**

Mental health problems among adolescents is a global challenge. During the transition to adolescence, physiological, psychological, and social changes occur, leading to increased vulnerability. Thus, adolescent boys are less likely to seek help for mental health problems, which makes them an undetected group. The aim of this study was to gain a deeper understanding of adolescent boys’ experiences of mental health and school health service.

**Methods:**

An inductive, qualitative design was chosen using three focus group interviews and three individual interviews. The study included 18 adolescent boys in 7th grade, in a school located in a medium-sized municipality in Norway. The interviews were analysed with qualitative content analysis. The consolidated criteria for reporting qualitative research (COREQ) were followed in this study.

**Results:**

The overall theme “barriers towards seeking help”, and three categories— navigating stigma and privacy concerns; perceptions of self-responsibility; and lacking knowledge of mental health problems and help-seeking—described the adolescent boys experiences. The awareness and willingness to seek help were present, but there are barriers preventing the adolescent boys from acting on that willingness.

**Conclusions:**

Lack of knowledge and a non-permissive culture for mental health problems among adolescents contributes to decreased help-seeking behaviour among adolescent boys. The school health service is the most related health service for adolescents and should focus on being available and strengthening empowerment and mental health literacy through the development and implementation of interventions to promote mental health.

## Background

Globally, mental health problems are increasing, and approximately 14% of all adolescents are affected [1]. About 50% of all mental health problems start before the age of 14 [2, 3], and mental health disorders typically manifest between ages 12 and 1870% manifest before 18 years of age [1, 2]. It is crucial to address early detection and treatment [1], as suicide is one of three leading causes of death among adolescents [3]. Adverse childhood experiences are associated with a two-fold increase in the risk of common mental health problems or suicidality in later life [1, 4, 5]. The World Health Organisation (WHO) defines adolescence as ages 10–19 years [6]. During this transition from childhood to young adulthood, major physiological, psychological, and social changes occur, leading to increased vulnerability and demands for adaptation [7]. Mental health is an integral aspect of the overall well-being, encompassing emotional, psychological, and social dimensions [1, 6]. Mental health is defined as “a state of mental well-being that enables people to cope with the stresses of life, realize their abilities, learn well and work well, and contribute to their community” [8]. The mental health of adolescents is affected by a combination of several factors. The most common types that can negatively affect health and well-being are childhood maltreatment (sexual, physical, or emotional abuse), maladaptive parenting, and bullying [5, 9]. During adolescence, there emerges a disparity in the health conditions of adolescent boys and adolescent girls, and this incongruity continues into adulthood [10]. Mental health problems such as anxiety, depression, or an eating disorder is more common among adolescent girls, whereas adolescent boys face a higher likelihood of experiencing suicide or being diagnosed with behavioural disorders like attention deficit hyperactivity disorder (ADHD) [11, 12]. Mental health promotion should include early detection and protective and supportive environments for mental health which can include both family and the school [1, 13].

School health services play a key role in health promotion and preventive mental health work in the municipality and work actively to strengthen the mental health competence of children and young people [14]. In Norway, school nurses play an important role in the field of public health and are expected to promote good health, provide health education, and address health problems in the adolescent population [15]. Granrud et al. [16] states that public health nurses generally feel that they have the necessary expertise to meet the needs of the adolescents who seek them out. However, a recent study by Putkuri and colleagues [17] described the competency of public health nurses in school health services and showed that it was insufficient and defined largely by good interaction skills. Competency regarding mental health problems among public health nurses needs to be developed to meet the requirements of the work.

Empowerment can be defined as a process where individuals, groups, or communities mobilise resources to take control of factors that affect their health [18]. Mental health literacy is related to empowerment and originates from the field of health promotion research [19, 20]. Kutcher et al. [19] defines mental health literacy as “understanding how to obtain and maintain good mental health; understanding mental disorders and their treatments; decreasing stigma related to mental disorders; and enhancing help-seeking efficacy (knowing when and where to seek help and developing competencies designed to improve one´s mental health care and for self-management capabilities)”. A recent pilot study [21] that developed a psychoeducational intervention for adolescents showed improvement in knowledge, skills, and behavioural intentions, and highlighted the important role of mental health and the value of empowering adolescents to mental health literacy in schools. In general, adolescent boys affected by mental health problems such as emotional distress, are less likely to seek help and avoid health services to a greater extent than girls, due to masculine norms and poor mental health literacy, making them an undetected group [22–24]. Studies shows that it was easier to seek help at a later stage when there has been a good experience of seeking help early in life [16, 25], and it would therefore be beneficial to strive to give adolescent boys the experience of seeking help as early as possible in life. Mental health literacy can empower the community to act for better mental health. Improvements in mental health literacy among adolescents have the potential to increase the ability to identify when, why, and how to seek help [26].

Previous research has focused on the school nurse’s perspective of mental health [27–29], but also on adolescent boys who had visited the public health nurse because of mental health problems [16], and adolescent males who had not received help for psychosocial problems from any formal help services [30]. According to adolescent boys (16–21 years old) who have been in contact with school health services, the first barrier to seeking help is also the largest [16]. Due to an increased prevalence of mental health problems [1, 6], and since school health services involved in preventive mental health work [14, 28] need to pay attention to factors that can affect the health of the adolescents [31], it is important to listen to the voices of adolescents about their experiences of mental health and school health services. However, to the best of our knowledge, this is the first study that includes experiences of adolescent boys in 7th grade, who either have or have not visited the school nurse for mental health problems. Therefore, the aim of this study was to gain a deeper understanding of how adolescent boys experience mental health and the school health service. This aim was specified through the following research question:

What factors hinder or enable adolescent boys from accessing school health services for mental health issues?

## Methods

### Design

The study used a descriptive and explorative qualitative design with an inductive approach [32], utilising both focus group interviews and individual interviews. The study adhered to the consolidated criteria for reporting qualitative research (COREQ) [33].

### Setting

School health service in Norway consists primarily of public health nurses also referred to as ‘school nurses’, who are nurses with a master’s level university degree in nursing. School nurses in Norway have a bachelor’s degree in nursing and one year of further education in clinical nursing i.e. school nursing. Until 2024 it has been optional to complete the master’s degree, with one more year of further education. From 2024 the further education is a full master’s degree. In addition, other professional groups are linked to the school health service, with variations between municipalities; mainly physicians, psychologists, and physiotherapists, but also other professional groups [34]. The school nurse in the school health service is responsible for children aged 5–20. The study was conducted in a medium-sized municipality on the west coast of Norway from November 2020 to January 2021. Out of 16 schools in the municipality, 4 schools were considered to have a large enough number of pupils to merit recruitment. Three of these schools were excluded because of the first author’s affiliation. The final recruitment was conducted from a school with an approximate of 320 pupils.

### Sampling and data collection

The study employed a purposive sample, meaning that participants were selected based on specific criteria relevant to the research objective [35]. The inclusion criteria were adolescent boys (AB), in 7th grade, who either have or have not used the school health service. No exclusion criteria were established. All adolescent boys in grade 7 at the included school were invited to participate in the study. The sample consisted of 24 adolescent boys. Of these, 19 adolescent boys aged 12 years, from one school, agreed to participate in the study. Due to the illness of one participant, a total of 18 participants were interviewed. There were three options in the consent form: either only focus group interviews or only individual interviews or both, which was communicated verbally and in the information letters to participants and parents.

Information about the project was given orally by the first author (THL), together with a written information letter to the participants. At the same time, the participants were given information letters and consent forms to their parents. The participants were informed that not everyone would be interviewed individually, even if they agreed to individual interviews. The participants for the individual interviews were selected based on statements that the first author wanted to explore in depth or ask further questions about. Signed consent forms were placed in a locked mailbox that was left in the classroom. Seventeen participants participated in focus group interviews, of which two participated both in focus groups interview and individual interview. In addition, one participant only took part in an individual interview.

Both focus groups and the individual interviews were based on a semi-structured interview guide and the opening question was “Do you know how to contact the school nurse?” Other questions included “What are your thoughts on the concept of mental health?”. The interview guide included some specific questions about the participants’ knowledge of the school nurse and their role in the school, such as “The school health service should work for good health among the pupils at the school. What do you think the school nurse can do for them?” and “What must the school nurse do to enable you to talk about what is important to you?” and “What challenges may exist with visiting the school health services?”. Follow-up questions and prompts were used, to a greater or lesser extent, throughout the entire interview. Data collection persisted until saturation was attained, indicating that no new substantial information surfaced from subsequent interviews. All digitally recorded interviews were transcribed verbatim by the first author (THL).

#### Focus group interviews

Three focus group interviews were conducted during one-week intervals, with 5–7 participants in each group. The focus group interviews were conducted in a room at Ung Arena, in a medium municipality in Norway, a leisure facility for young people in the municipality, where various support services are available (e.g., youth health centres, psychologists, social workers). The intention was to create a safe conversational environment for participants to ask questions of greater depth, mainly with the use of open questions, such as ‘what’ and ‘how’ questions [35]. The first author (THL) was the moderator (M). A public health nurse colleague was involved as a secretary and was not acquainted with the participants or the school. The semi-structured interview guide developed for this study was used. The interviews lasted between 23 and 48 min.

#### Individual interviews

Three individual interviews were conducted by the first author (THL), whereof one of the participants attended only in the individual interview due to an inability to attend the planned focus group interview. The other two participants were included based on statements made in the focus group interviews which researchers wanted to explore further. Before the interviews began, the participant received information about the aim of the study, confidentiality and voluntary participation, and the right to decline participation at any time. The semi-structured interview guide was applied during the interviews. However, the individual interviews made it possible for the participants to extend and reflect on the content of the focus group interviews, which contributed to the use of the questions in a slightly different way: “At the focus group interview you stated…” and “Tell me more about why you think it is like that…”. The interviews lasted between 12 and 18 min.

### Data analysis

The transcribed interviews were inductively analysed using qualitative content analysis developed by Graneheim and Lundman [32] to identify emergent themes and patterns in both manifest and latent content [36]. The transcripts were read through several times by three of the authors (THL, ASL, IKH) to gain an understanding and to create an overall picture of the data. The analysis was carried out by the first author (THL), together with an ongoing discussion with two other authors (ASL, IKH) throughout the whole process to reach consensus. First, meaning units were identified for e.g., words, sentences, or paragraphs that represent content related to the aim of the study. Next, these units were condensed to shorten the text and then labelled with a descriptive code close to the original text in order to maintaining the manifest content. Furthermore, codes with similar meanings were then collected into different groups into subcategories and later categories. The categories were then compared, and patterns were sought which may indicate latent content, a theme. As a final check to confirm of the analysis, one of the authors (H-Å.P) took part in the analysis process by reading all the transcripts and the interpretations of the findings. This process lasted until agreement between all authors was reached. Trustworthiness was upheld by means of investigator triangulation involving several researchers [37]. The participants’ names were pseudonymised to ensure confidentiality. To elaborate on the categories and make the participants’ own voices clear, quotations are used in the results. Examples of the analytical procedure are given in Table 1.

**Table 1:** Example of the analysis process

Meaning unit	Condensed meaning unit	Code	Subcategory	Category	Theme
Maybe, take out, so that everyone gets taken out once a year approximately, to talk to the school nurse. Because it can be a bit difficult for some to go to the school nurse alone, and then everyone sees it, and they might not want that. Then surely everyone will come and ask, why were you there? And then you don’t want to say it.	Consider scheduling annual check-ins with the school nurse for everyone to address any concerns privately, avoiding potential discomfort of going alone and facing questions from peers.	Easier to go if others get it too	Protecting themselves	Navigating stigma and privacy concerns	Barriers towards seeking help
Girls break up for a long time, while boys make up. They can be unfriendly for 2–3 years. But if we’re experiences the same. Unfriended… half an hour later… friends again. There’s just something magical about boys. One day everything is fixed. No one cares anymore and everyone plays together.	Girls hold grudges for longer, while boys reconcile quickly. Despite experiencing similar conflicts, boys effortlessly repair relationships and continue playing together.	It is only among girls that it does not go away by itself	Mental health problems are not part of ‘boy culture’	Perceptions of self-responsibility	Barriers towards seeking help

## Results

The analysis resulted in one theme, three categories, and six subcategories (see Table 2). Adolescent boys’ experiences of mental health and school health services were abstracted into the theme “barriers towards seeking help”. Despite recognizing the importance of seeking help, the adolescent boys encounter significant obstacles that prevent them from taking action. These barriers include societal stigma surrounding mental health problems, a sense of self-responsibility that may discourage seeking help, and a lack of comprehensive knowledge about where to turn for help. As a result, even though adolescent boys may feel a desire to discuss their struggles, various factors hinder their ability to reach out for resources, leading to a gap between recognition of the need for help and seeking it.

**Table 2:** Adolescent boys’ experiences of mental health and school health services captured in one theme, three categories, and six sub-categories

Theme	Barriers towards seeking help		
Categories	Navigating stigma and privacy concerns	Perceptions of self-responsibility	Lack of knowledge about mental health and help-seeking
Sub-categories	*Protecting themselves*	*Mental health problems are not part of ‘boy culture’*	*Do not know who to go to for what*
	*Protecting close ones*	*You have to tolerate it*	*The school nurse must be known*

### Navigating stigma and privacy concerns

There was a strong need among the adolescent boys not to let others know whether they sought help. They wanted to protect themselves and others and were reluctant to let anyone know or ask them questions about visiting the school nurse. This reluctance stems from a combination of navigating stigma and privacy concerns surrounding mental health problems. The adolescent boys felt a sense of vulnerability in seeking help, fearing judgment or negative perceptions from their peers. Additionally, they may have wanted to shield their close ones from worrying about them or facing potential stigma associated with mental health problems.

#### Protecting themselves

The adolescent boys described that there was usually always one or several ways to reach the school nurse, which most often involves asking the teacher. Thus, they expressed a worry about what questions the teacher would ask when they asked to visit the school nurse. They struggled to avoid being taken out of the classroom by the teacher and get attention and it felt easier to leave if none of the other students knew where they were headed. The adolescent boys did not want the other students to know that they went to the school nurse, because a lot of questions would arise about the visit, and it would be uncomfortable to answer these questions. In addition, they were afraid of what the other students would think of them. The adolescent boys said that it would facilitate them to seek help if they could book an appointment in advance with the school nurse; for example by the phone. Previously, the classroom was located next to the office, which was an advantage and made it easier to make discrete visits. Concerns about what parents would think if the adolescent boys told them they had visited the school nurse were also highlighted. Feelings emerged, such as the fear that the parents would ask why they had shared this with the school nurse. Another fear that was highlighted was that what they told the school nurse would follow them for the rest of their lives if anyone found out, so they preferred to avoid telling others about their problems.

*AB1: It’s not so easy to say to the teacher “my father is abusing me”, it’s better to go down to the school nurse, because she works with it.*

*AB2: But if the teacher asks, you can just say “no, personal reasons”.*

*AB1: Yes, you can just do that.*

*AB3: But then the whole class starts talking about it. Like “have you heard what he said”.*

*M: Okay, so sometimes it can be a bit silly to have the teacher as an intermediary?*

*AB1: Yes, should have had a note in the classroom saying when she’s at school, so you know when you can come down.*

*AB4: Yes, and the number.*

*AB1: And maybe it could say “the school nurse can help you with everything” in some way. Not just injuries. Because when you hear nurse, you hear sick or injured in a way.*

*AB3: I think it’s a good idea to say what she can help with. (FG [Focus group] 1)*

#### Protecting close ones

The adolescent boys emphasised a fear of being asked about what they had talked about during their visit to the school nurse, partly because they did not want to share, but also because it was difficult to say it. In addition to protecting themselves, they also wanted to protect close ones, as they were clear that they did not want to transfer negative experiences or concerns to friends or family. The adolescent boys mainly felt that they wanted to protect their families, both from worry and from feeling that they were revealing something personal that should have been handled at home. At the same time, they expressed wondering whether their parents thought it was a good idea to talk to someone outside the family. This contributed to the fear that difficulties will increase if they speak about things like conflicts with family or friends, changes in relationships with teachers, or other uncomfortable situations.

*M: Yes, and what’s wrong with everyone knowing that you’re not doing very well now?*

*AB1: Then everyone will feel so sorry for you, and you don’t really want anyone to know anything.*

*AB2: If you’re having a hard time, something more difficult will come up.*

*AB3: That you don’t want more people to get into that difficult situation. You want them to live life.*

*M: Because if someone is sad, it can spread to the others?*

*AB2: Yes, if your dog dies, your friend may be sad because they enjoyed being with the dog. So, you just want to…*.

*M: Protect?*

*AB2: Yes, they will comfort on your behalf, maybe, you know.*

*M: I think you may be right that we protect those around us.*

*And that is a good quality. (FG 3)*

### Perceptions of self-responsibility

The adolescent boys were clear and aware that adolescent boys and men also can suffer from mental health problems and disorders, and that it is nothing unusual. This illustrates an internal conflict between awareness of the need for help and a sense of self-responsibility to address their own problems. However, their perceptions of self-responsibility, coupled with the belief that mental health problems are not part of ‘boy culture,’ present significant barriers to seeking help. They feel a sense of obligation to handle their issues on their own and may even believe that they must tolerate these problems.

#### Mental health problems are not part of ‘boy culture’

The adolescent boys felt that mental health problems were seen as ‘drama’ and ‘girl stuff’ and that problems that are not visible were perceived as drama and something they choose to not focus on. Furthermore, they expressed that they have a good culture with no conflicts, and they put things behind them—unlike girls. At the same time, they were clear that adolescent boys also have mental health problems and gave several concrete examples. But they also expressed that they did not want to be open about it if they have mental health problems or thoughts of suicide because it is not something that is part of the culture. They said that they do not want anyone to feel sorry for them, because then attention is directed at them, and they are associated with these problems. These contradictions create major barriers to seeking help, and a description of how they just hope it will pass is expressed. A feeling of shame is noted and that it does not go away because it is identified as ‘girly’.

*M: Yes, so you are very aware that boys can also have difficulties?*

*AB3: Yes, but it’s not the coolest thing for a boy to say. That I have thoughts or want to commit suicide.*

*AB4: For example, boys say: you’re so bad or something like that. And then I don’t say much. But whoever hears it then takes it pretty hard.*

*AB3: Yes, and we boys, it’s kind of like I said, it’s not the coolest thing to go to the school nurse and talk about it. But everyone kind of wants to do it, but…yeah. (FG1)*

#### You have to tolerate it

The adolescent boys explained that they must deal with both negative comments and negative emotions. However, they point out that there are several comments from friends in the good culture, which are not necessarily meant to be unpleasant, but that it is not pleasing if it is something they have experienced themselves. The friends could joke about the mental health problems, which was not appreciated by the adolescent boys. To divert their thoughts, the adolescent boys used, for example, games or similar, while they experienced the discomfort of feeling it, or talking about it, as a burden. They express that they did not want to remind themselves of how they really feel.

*M: We’ve talked about the fact that some people find out, but what other things do you have in mind?*

*AB1: Because I think boys make more fun of things than girls. For example, if you have ADHD, boys joke about it more. Because it’s like that, and it’s not so much fun to get it out.*

*M: Because it can easily be joked about.*

*AB1: Yeah. In many different ways. Stupid fool, nice fool. Or just plain rude.*

*M: So what can be a nice fool?*

*AB1: I don’t really know.*

*M: Maybe it depends on how you feel. (FG 2)*

### Lack of knowledge about mental health and help-seeking

The adolescent boys described how difficult it is for them to assess what is bad enough to seek help and what is worth seeking help related to lack of knowledge. Due to difficulties talking about the problems, it is better to hope that it will pass by itself. This lack of knowledge about mental health and help-seeking further complicates their decision-making process. The adolescent boys express a desire to seek help, recognizing the importance of addressing their issues, yet they face barriers such as not knowing who to go to for what. In this context, the school nurse must be known as a resource.

#### Do not know who to go to for what

The adolescent boys believe that they need to have a very good reason to go to a school nurse, and they do not know when the symptoms become serious enough that they should seek help. They were clear that some problems belong to the family, some are the responsibility of the teacher, while others should be discussed with the school nurse, like when it comes to mental health. However, they are not as clear about which problems belong to which side. Due to the discomfort in discussing mental health problems with unfamiliar individuals, such as the school nurse, adolescent boys seek further information. They desire clarity on the school nurse’s role, how she interacts with students, and the types of help and support available. The adolescent boys stated that they would like to see the school nurse more often, generally in the school environment, but also in the classroom. They gave examples of how the school nurse could be more visible, such as by giving some lessons on mental health in the classroom. If they become accustomed to seeing the school nurse, they think it will be easier to approach the person.

*AB1: We have a very good relationship with our teachers then. So, we can talk to them too.*

*AB2: The school nurse might be a bit…It may be easier to talk to friends or parents. Because we do not see the school nurse very often, it is not that easy…You don’t really know the person who is there. A bit like talking to, it feels like a stranger.*

*AB1: We know who it is, but we do not know her.*

*M: And then it is a bit difficult to talk about how you feel?*

*AB3: Yes*

*M: I can understand that. How do you wish you could contact the school nurse then?*

*AB3: Just go downstairs then. Nothing worse than that. Or if you do not want anyone to know, just book an appointment or something. (FG 2)*

#### The school nurse must be known

The adolescent boys thought that it is easier to visit the school nurse when the person is known, and when she knows the students. Otherwise, they felt like they were talking to a stranger. The adolescent boys described a concern about how the school nurse encountered them in help-seeking situations. They needed to know that it is okay to have different reactions when they visit a school nurse and talk about feelings. It should be a permissive climate for showing feelings like crying. There were also descriptions concerning the fear of being trivialised, laughed at, or that the school nurse will pass on the information the adolescent boys shared. They want the school nurse to show that they take their problems seriously. Concrete tools for how to handle issues were requested by the adolescent boys, and a call for support from the school nurse to find a solution was addressed. The adolescent boys wanted the school nurse to be more accessible. The only opportunity to visit the school nurse without intermediaries is to drop by her office. Thus, if the adolescent boys are unsure whether she is in the office or has time for them, it is easier not to see her.

*M: But what should he or she do to enable you to talk about what’s important then?*

*AB2: Being able to trust them.*

*AB3: Listening.*

*AB4: Not telling everyone.*

*AB5: Having to do something about it.*

*AB2: Maybe we should have a school nurse…who might be in the class or something like that. Get more used to her in a way. The only time we’ve seen her is when we’ve taken injections. Or we’ve hurt ourselves.*

*AB4: Those are the two times we see the school nurse.*

*AB5: Or if she’s going to say something.*

*AB2: It should be more like the nurse should perhaps be a bit more social, so that the students see her more often.*

*AB1: A little more active. (FG 1)*

## Discussion

In this qualitative study, adolescent boys describe their experiences regarding mental health and school health services, which is abstracted as “barriers towards seeking help”. The findings in this study describe several experiences and feelings of adolescent boys, revealing their struggles with opening up about mental health problems, overcome barriers and utilizing available resources.

In this study, stigmatisation, and the fact that mental health is not part of ‘good’ culture, is portrayed as a barrier to seeking help. Stigma is the most frequently cited barrier in studies related to help-seeking behaviour for mental health problems among adolescents, according to systematic reviews [25, 38]. Stigmatisation can be understood as a combination of factors such as stereotypes, prejudices, and discrimination, and means “to label”, and figuratively it is used to label someone negatively in a social context [39–41]. Stigma can increase in line with lack of knowledge and is connected with negative attitudes and can prevent individuals from making good choices for their own and others’ mental health [19]. Reducing stigma is a key part of mental health promotion and should be a focus area for school health services, both in terms of help-seeking, but also concerning mental health in general [1]. The adolescent boys expressed that they must fend for themselves and tolerate mental health problems; it is part of a kind of ‘good’ boy culture that defines girls as having mental health problems, but not boys. These attitudes are also described by Granrud and Bisholt [16], who point out that in addition to fearing being seen as feminine, the adolescent boys also feared being seen as ‘weak’ or ‘crazy’. In this study, the adolescent boys are ambivalent, as they know that mental health problems also can affect adolescent boys, but it is not considered masculine to discuss them. Adolescence is a period for boys that includes masculine identity formation and social conformity clashes that promote risk-taking and denial of feelings of vulnerability [42], which is in line with the adolescent boys´ descriptions that they want feelings to pass, to avoid being reminded of how they really feel. This need for independence has frequently been mentioned in research [43], and has been identified as a barrier to mental health help-seeking [44]. With more knowledge, the hope is that stigma can be reduced and the factors that cause adolescent boys to feel ashamed and avoid talking about what is difficult, can be nuanced. School-based interventions with focus on stigma reduction may be of great value when it comes to help-seeking for mental health [45, 46]. Efforts to reduce barriers can increase adolescents’ empowerment, which can contribute to their ability to make good choices for the benefit of their own and others’ mental health, which in turn will be of great importance when the adolescent boys need to seek help. At the pupil’s level, empowerment entails educating adolescent boys about mental health, breaking the stigma surrounding seeking help, and offering support structures within schools that foster openness and participation.

The findings from this study show that there is a need for more knowledge about mental health among adolescent boys. They report that they want school nurses to be visible, informative, proactive, and trustworthy. They are unsure whether different problem areas belong to teachers, parents, friends, or school nurses, and could not determine when it is serious enough to talk to someone about their problems. Not knowing where to seek help has also been described in the literature [25, 38, 47] as one of the most frequently cited barriers for children and adolescents when it comes to seeking help for mental health. One of the mission of school health services, is to deliver health promotion and prevention in relation to physical, mental and sexual health [15, 48]. School health services should be aware of barriers and focus on facilitating factors to increasing the knowledge of children and adolescents, and should work actively to strengthen the mental health competence of children and young people [14]. In this study, the adolescent boys explained that they wished the symptoms would go away on their own, thus it is difficult to deal with the symptoms, which leads to distracting themselves by playing games or other activities. This indicates that adolescents need more knowledge about what mental disorders entail and what types of treatment that are considered, which is encompassed as a part of promoting mental health competency [19, 43, 49, 50]. Making good choices for the benefit of one’s own health is also one of the principles underlying empowerment [15, 48], and school health services are and should be central in this work [51]. Desire is an empowering factor, and school nurses should focus on increasing adolescents’ knowledge about mental health so that they can utilise this resource. Many of the experiences and feelings during childhood and adolescence are related to the normality of being a young person in development [42]. At the same time, it is during adolescence that most mental disorders begin [1, 6], which is reason enough to stress increased knowledge about mental health as early as possible during adolescence. WHO [6] claims that all children and adolescents should be given the opportunity to achieve necessary skills and competences about health to be able to have a healthy transition from childhood to adolescence and from adolescence to adulthood. Mental health promotion skills must be tailored to the recipient so that they are concrete and relevant. The same type of training cannot be given to adolescents as to adults [18]. For adolescents, mental health promotion skills can increase their knowledge and understanding, reduce stigma, and enable them to make good choices for their own and others’ mental health, including knowing when, where, and how to seek help for mental health problems [18]. On an individual level, empowerment means bolstering each adolescent boy’s sense of self-confidence and self-esteem and providing them with the skills needed to take care of their own mental health and seek support when needed.

The adolescent boys expressed that they did not want others to know that they sought help from the school nurse, which was influenced by the boys’ culture, and there was also a lack of knowledge about mental health and how to seek help for mental health issues. Related to this it is vital to address the barriers experienced among adolescent boys in connection to mental health and school health services, which can contribute to achieving the Sustainable Development Goals (SDG), especially SDG 3 [52]. Although the onset of most mental disorders occur during childhood [4, 5], effective treatment is typically delayed due to the absence of identified needs among adolescents, and lack of help-seeking behaviour. One reason for this can be the fact that school health services often lack specialised knowledge and age-appropriate referral sources to identify the early signs of mental health problems.

To address this concern, the study’s findings repeatedly highlight the limited interaction with school nurses and suggest the possibility of facilitating contact through teachers. In light of this, the clinical implications of our findings emphasize the importance of early identification and intervention development as essential strategies for addressing adolescent boys’ mental health problems. Furthermore, by the identification of barriers that exist to accessing school health services, the understanding of how to develop and implement targeted interventions to strengthen mental health literacy among adolescents can be more effective and equitable. Further research should focus on school health services’ perspectives on how to develop and implement school health services’ interventions promoting adolescents’ mental health literacy.

### Strengths and limitations

All interviews were conducted with the first author, who has experience in school health care through the profession as a public health nurse. In addition, among the other authors, the preunderstandings include nursing in both paediatric and public health, and experience in qualitative research, which was a strength when interpreting the results. Integration of researchers with different educational backgrounds and different pre-understanding can be considered as a strength due to various reflections and interpretations [53]. Throughout the process, the authors have strived to be aware of their own preunderstandings [35]. An awareness of the pre-understanding through all the interviews were employed, to prevent that the follow-up questions should be influenced. Similarly, discussions and reflections with co-authors throughout the analysis process contributed to preventing interpretations from being influenced by pre-understanding. This action taken to prevent influence of pre-understanding could strengthen the dependability of the findings [35, 37]. Investigator triangulation was also applied, involving several authors to best ensure the trustworthiness of the results [37]. Having several authors contributes to strengthening the credibility of the findings [35, 54].

The choice to employ a purposive sample could strengthen the study’s credibility because it allows researchers to target individuals who could provide relevant and insightful information related to the research question. A purposive sample can be particularly useful when studying specialized populations or phenomena where specific expertise or experiences are required [35, 37].

The intention of including both focus groups and individual interviews as a data collection method was to involve more participants, meet individual preferences and investigate if there were other statements that approached in individual interviews than in focus groups. Talking about mental health with participants at this age can be a sensitive subject [48]. One of the data collection methods applied in the study was focus groups, which can be seen as limiting when it comes to capturing experiences when discussing a sensitive topic [35]. However, focus groups can have several benefits, as they provide insights into complex tasks and may provide perspectives that are difficult to obtain from individual interviews [55]. During data collection, it became clear that the participants in the focus group interviews were active and spoke freely. The content was also fuller and broader than in the individual interviews, where the participants to a greater extent only answered specific questions from the interviewer. In this way, it was felt that the focus group interviews gave the participants strength and confirmation when they talked about difficult issues. It may be difficult for the researcher to create the same confidence in individual interviews [55].

When a recurring pattern emerged in the data, experiences, statements, or words were repeated without anything new being added, was the guarantee of the achievement of data saturation, rendering further interviews unnecessary to fulfil the study’s objectives.

A limitation is that all participants came from the same school, and contextual factors may differ in other schools, which may limit the study’s transferability [35], which needs to be considered. The authors chose the school where the first author had no personal interest in and considered that it had enough participants when the sampling was done. However, there were a mix of participants who both had, and had not visited, the school health service, which can be seen as a strength when it comes to catching both perspectives. By interviewing them early in their adolescence, it was hoped that the adolescent boys would be interviewed before any of their ailments or disorders arose. The availability of school health services/school nurses will vary between municipalities and countries, and the study’s transferability must be seen in this light.

## Conclusion

School health services play a key role in working with mental health but despite this, several barriers exist such as stigma, an unaccepting culture among adolescent boys regarding mental health problems, and a lack of knowledge. The barriers the adolescent boys described can be connected to a lack of mental health literacy and insufficient school health services. To enable adolescent boys to increase their knowledge of how to promote and maintain good mental health, and thus better prevent problems and disorders, systematic efforts to strengthen mental health promotion skills needs to be integrated in school health services. Empowerment at various levels is crucial in promoting mental health among adolescent boys. Empowering adolescents can give them control over factors that affect their health and increase possibilities of help-seeking. An important element of mental health promotion is reducing stigma, which is central for adolescent boys at this age. School health service, which are the most relevant and accessible health services for adolescents, should actively focus on an early integration by providing knowledge needed to facilitate help seeking for mental health problems. Further research needs to be directed towards school health service interventions that promote adolescents’ mental health literacy.

## Data Availability

The datasets used during the study are available upon reasonable written request, in accordance with ethical approval and with permission of Lund University.

## Abbreviations

AB: Adolescent boy
FG: Focus group
M: Moderator
WHO: World Health Organisation
